# Application of Image-Based Phenotyping for QTL Identification of Tiller Angle in Rice (*Oryza sativa* L.)

**DOI:** 10.3390/plants13233288

**Published:** 2024-11-22

**Authors:** Yoon-Hee Jang, Song Lim Kim, Jeongho Baek, Hongseok Lee, Chaewon Lee, Inchan Choi, Nyunhee Kim, Tae-Ho Kim, Ye-Ji Lee, Hyeonso Ji, Kyung-Hwan Kim

**Affiliations:** 1 Department of Agricultural Biotechnology, Gene Engineering Division, National Institute of Agricultural Sciences, Rural Development Administration, Jeonju 54874, Republic of Korea; uni315@korea.kr (Y.-H.J.); greenksl5405@korea.kr (S.L.K.); firstleon@korea.kr (J.B.); knh702@korea.kr (N.K.); jhs77@korea.kr (H.J.); 2 Department of Southern Area Crop Science, Crop Production Technology Research Division, National Institute of Crop Science, Rural Development Administration, Milyang 50424, Republic of Korea; ehg117@korea.kr (H.L.); 3 Department of Central Area Crop Science, Crop Cultivation & Environment Research Division, National Institute of Crop Science, Rural Development Administration, Suwon 16613, Republic of Korea; wowlek44@korea.kr (C.L.); yjlee0@korea.kr (Y.-J.L.); 4 Department of Agricultural Engineering, Division of Smart Farm Development, National Institute of Agricultural Sciences, Rural Development Administration, Jeonju 54874, Republic of Korea; inchchoi@korea.kr; 5 Department of Agricultural Biotechnology, Genomics Division, National Institute of Agricultural Sciences, Rural Development Administration, Jeonju 54874, Republic of Korea; thkim1961@gmail.com

**Keywords:** rice, tiller angle, quantitative trait loci (QTL), phenotyping, Red-Green-Blue (RGB), parameter

## Abstract

Rice tiller angle is a key agronomic trait that regulates plant architecture and plays a critical role in determining rice yield. Given that tiller angle is regulated by multiple genes, it is important to identify quantitative trait loci (QTL) associated with tiller angle. Recently, with the advancement of imaging technology for plant phenotyping, it has become possible to quickly and accurately measure agronomic traits of breeding populations. In this study, we extracted tiller angle and various image-based parameters from Red-Green-Blue (RGB) images of a recombinant inbred line (RIL) population derived from a cross between Milyang23 (*Indica*) and Giho (*Japonica*). Correlations among the obtained data were analyzed, and through dynamic QTL mapping, five major QTLs (*qTA1*, *qTA1*-1, *qTA2*, *qTA2*-1, and *qTA9*) related to tiller angle were detected on chromosomes 1, 2, and 9. Among them, 26 candidate genes related to auxin signaling and plant growth, including the *TAC1* (*Tiller Angle Control 1*) gene, were identified in *qTA9* (RM257-STS09048). These results demonstrate the potential of image-based phenotyping to overcome the limitations of traditional manual measurements in crop structure research. Furthermore, the identification of key QTLs and candidate genes related to tiller angle provides valuable genetic insights for the development of high-yielding varieties through crop morphology control.

## 1. Introduction

Rice architecture is an important agronomic characteristic that affects yield, determined by the size and shape of the leaves, stems, and panicles [1]. Tiller angle is a key factor in shaping plant architecture, as it determines how the leaves of the rice are arranged and how effectively they can absorb sunlight [2]. Depending on the tiller angle, the photosynthetic efficiency, planting density, and air circulation of rice are greatly affected [3]. For example, rice plants with a wide tiller angle have a spread-out structure, are less susceptible to pests and diseases, and can collect a lot of light, but they occupy too much space. A small tiller angle is vulnerable to pathogen attacks, but maintains an appropriate distance between plants to increase the yield per unit area [4]. Therefore, tiller angle needs to be optimized according to the growing environment of rice, and under certain cultivation conditions, an appropriate tiller angle can maximize crop yield.

For a number of years, genetic factors regulating tiller angle have been studied, and various QTLs and genes were identified. *TAC1* was identified as a major QTL for tiller angle in various studies [5]. *TAC1* on chromosome 9 is a key factor regulating tiller angle between *indica* and *japonica* rice [6]. *TAC1* encodes a 259-amino acid protein of unknown function, and mutations in the fourth intron of the 3′-untranslated region reduce tac1 expression, resulting in compact plant architecture [7]. Major QTLs *qTAC9* and *qTAC8* regulating tiller angle were identified in RIL populations derived from japonica rice D50 and *indica* rice HB277. *qTAC9* was mapped to the same locus as *TAC1*, and *ORF7* encoding a basic helix-loop-helix protein may be the underlying gene for the newly discovered *qTAC8* [8]. One major gene, *Ta*, and 11 QTLs are largely responsible for tiller angle variation in the Lemont/Teqing F_2_ population. *Ta*, identified on chromosome 9, and four QTLs (*QTa1*, *QTa2*, *QTa5*, and *QTa8*) account for 69.1% of the genotypic variation in tiller angle [9]. In Asian wild rice (*Oryza rufipogon*), which has a prostrate architecture, *PROG1* and *TAC1* were identified on chromosomes 7 and 9. *TAC1* is involved in tilting the first tiller, whereas *PROG1* is responsible for tilting the first and second tillers. The *PROG1* gene encodes a single C2H2 zinc finger protein, but the downstream factors and the detailed mechanisms by which they act are not yet known. In cultivated rice, disruption of *PROG1* function results in upright growth [10,11]. *qTA3* on chromosome 3, specifically explored in *indica* rice, largely determines the natural variation of tiller angle in rice, and the newly discovered *Tiller Angle Control 3* (*TAC3*) gene in this region is preferentially expressed at the base of the tiller and encodes a conserved hypothetical protein controlling tiller angle [12]. *LAZY1* (*LA1*) is the first identified gene to control tiller angle in rice and is located on chromosome 11. *LAZY1* affects the asymmetric distribution of auxin by regulating polar auxin transport, which controls the gravitropism of shoots and increases tiller angle in rice [13].

Despite the importance of tiller angle, existing methods for studying tiller angle have several limitations. Traditionally, tiller angle has been analyzed through direct observation and measurement by individuals. This approach is time-consuming and labor-intensive, making it particularly inefficient for large-scale experiments. Moreover, human measurements can be subjective, leading to challenges in obtaining consistent data, which may affect the accuracy and reliability of the results [14]. Recently, phenotyping technology has been attracting attention as a powerful tool to overcome these limitations. Phenotyping technology enables rapid acquisition of large volumes of plant images by utilizing various imaging information, and it has achieved significant growth with the development of high-resolution imaging equipment and image analysis algorithms, such as deep learning [15]. By using these technologies, plant phenotypes can be measured quickly and accurately, and vast amounts of data can be efficiently processed, making it a critical tool in crop breeding and precision agriculture [16]. In recent years, many research cases applying phenotypic analysis technology to tiller angle have been reported. Wu et al. analyzed the genetic structure of tiller growth by combining high-throughput micro-CT-RGB phenotyping and genome-wide association study [17]. The measurement of rice tiller angle using 3D digital structures of rice plants generated based on the location-separation-measurement method (LSMM) has an accuracy of more than 97% [18]. A web app integrating a trained CNN model and a Django server showed a counting accuracy of more than 99% between field-captured images and manual measurements [19]. Thus, phenotyping technology is particularly advantageous for large-scale data processing, and machine learning algorithms can be utilized to quickly process and analyze large amounts of data, making it suitable for large-scale QTL studies [20,21]. In addition, it can simultaneously analyze various phenotypic traits, providing a great advantage in comprehensive research on the structural characteristics of rice [22].

The aim of this study is to quantitatively evaluate the tiller angle of rice using image analysis techniques, facilitating a more accurate and comprehensive QTL analysis to identify the genetic factors influencing tiller angle. We measured accurate time-series data related to key agronomic traits associated with tiller angle and conducted large-scale QTL analysis to identify QTLs associated with tiller angle. Based on these results, we aim to provide new breeding resources for regulating tiller angle, which will support improvements in rice plant architecture suited to specific cultivation environments and ultimately contribute to increased yield.

## 2. Results

### 2.1. Standardized Growth Management for Phenotyping of Rice

**Figure 1:**
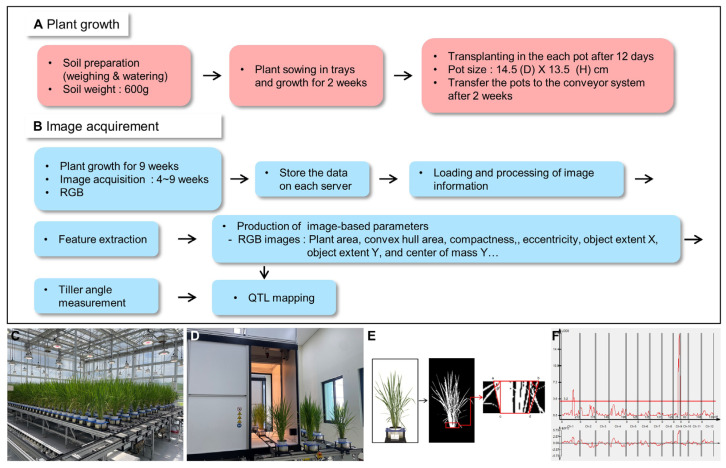
Workflow of image-based tiller angle measurement and QTL mapping in rice. (**A**) Plant preparation and growth in the greenhouse. (**B**) Image acquisition and feature extraction. (**C**) Plant growth in the conveyor system. (**D**) RGB imaging system. (**E**) Image-based automatic tiller angle measurement. a–d, coordinates assigned to the upper and lower vertices of the plant. (**F**) QTL mapping.

**Figure 2:**
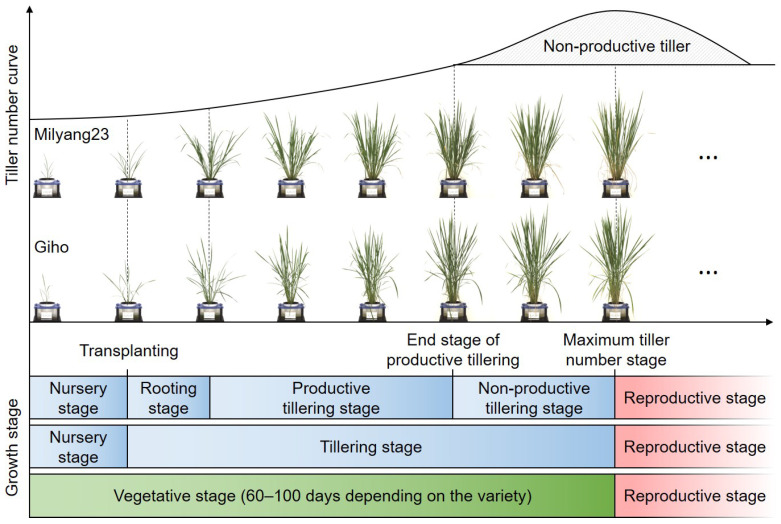
Developmental pattern of ‘Milyang23’ and ‘Giho’ during vegetative stages.

For accurate phenotypic analysis, rice was cultivated under stable environmental conditions. Milyang23, Giho, and the Milyang23/Giho recombinant inbred line (MGRIL) population were uniformly grown in the same amount of soil. The soil was flushed three times a day for three days to reduce salt damage, as salt reduces nutrient absorption and crop growth in the early stages. The rice plants were transferred to a conveyor system and imaged at consistent times and locations, with the data automatically stored on a server. RGB images were collected weekly from 4 weeks after sowing (W), when tiller formation begins in earnest after root establishment, up to the maximum tillering stage at 9W. Each line was planted with six individuals, and images were captured at three angles every week for 6 weeks, resulting in a total of 17,712 images. The acquired images were utilized to generate six image-based parameters to characterize the rice architecture and measure tiller angle, and the extracted data were used for QTL analysis (Figure 1). Figure 2 shows the tillering patterns of Milyang23 and Giho according to the growth stage. At 4W, the MGRIL population had 3–5 tillers, which steadily increased to a maximum of 27 by 9W. Subsequently, the late-formed weak tillers died off, leading to a structural stabilization of the plants and a decrease in tiller angle.

### 2.2. Tiller Angle Measurement and Its Changes Across Development Stages

**Figure 3:**
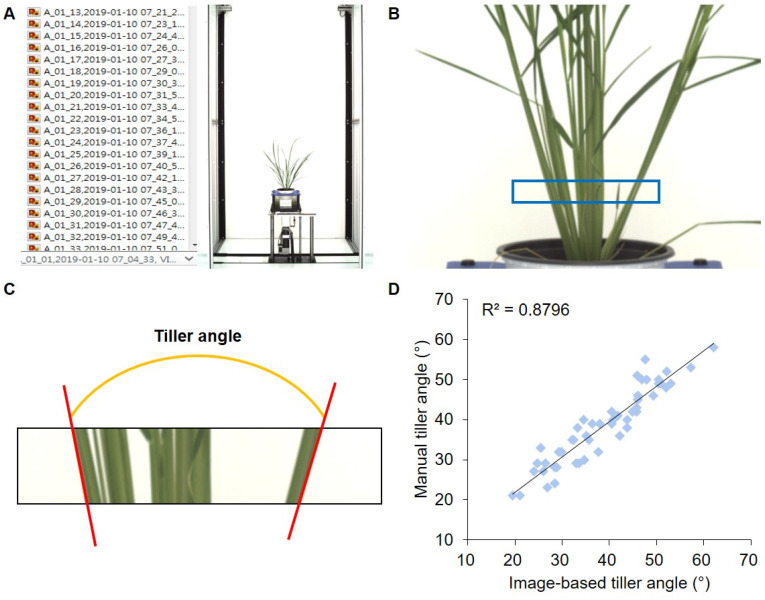
Program for automatic measurement of the tiller angle of rice. (**A**) RGB images of rice plants stored in the 3D scanalyzer imaging system server. (**B**) Measurement area recognition. The recognized area is marked with a blue rectangle. (**C**) Angle of tiller as measured by the program. (**D**) Correlation analysis between image-based tiller angle and manually measured tiller angle.

**Figure 4:**
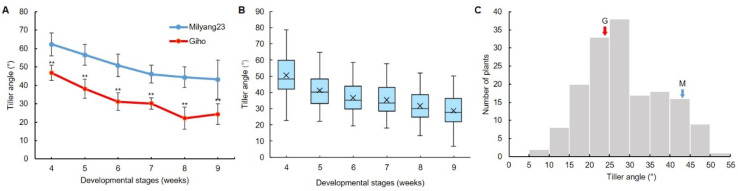
Variation and distribution of tiller angle by development stage. (**A**) Developmental pattern of tiller angle of Milyang23 and Giho. ** significant at the 0.01 level. (**B**) Box-and-whisker plot of tiller angle in MGRIL population. (**C**) Frequency distribution of tiller angle of MGRIL population 9 weeks after sowing. M, Milyang23; G, Giho.

The tiller angle was calculated using a tiller angle measurement program from RGB images (Figure 3A). Yellowing leaves were removed using differences in the Lab color space, and the tiller angle was calculated from images segmented to a specific height from the base of the plants (Figure 3B,C). To verify the accuracy of the program, the tiller angle was measured using 50 lines selected from the MGRIL population; a linear relationship was observed between the program-calculated values and the actual measurements, with a correlation coefficient of 0.8796 (*p* < 0.01) (Figure 3D). The variation and distribution of tiller angle of Milyang23, Giho, and the MGRIL population were analyzed across development stages (Figure 4, Table S1). The tiller angle of Milyang23 was always larger than that of Giho, and at 9W, the final tiller angle was 43.19° for Milyang23 and 24.37° for Giho, showing a difference of approximately 1.8 times (Figure 4A). The average tiller angle of the MGRIL population was 28.75° at 9W, which was lower than that of Milyang23 but higher than Giho (Figure 4B). The tiller angle calculated by the program showed a continuous distribution, confirming that it is a quantitative trait controlled by more than one multiple gene (Figure 4C).

### 2.3. Quantitative Analysis of Phenotypes Using RGB Image-Based Parameters

**Figure 5:**
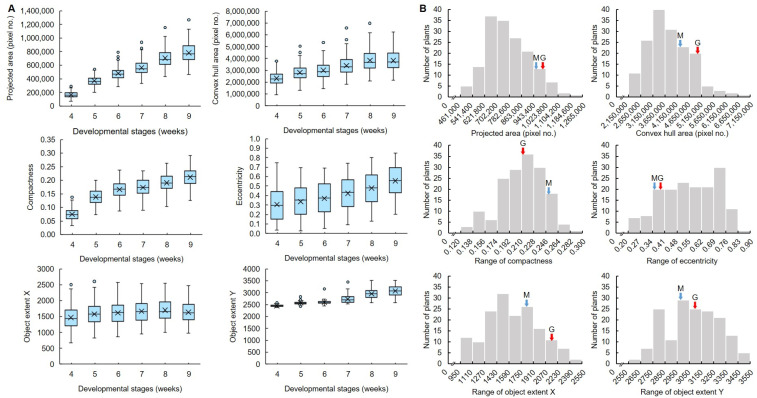
Analysis of the distribution of image-based parameters in MGRIL population. (**A**) Box-and-whisker plot of image-based parameters across development stage. (**B**) Frequency distribution of image-based parameters 9 weeks after sowing. M, Milyang23; G, Giho.

To identify traits related to tiller angle, six image-based parameters were extracted from RGB images of rice (Figure 5). As the growing period passed, the projected area, convex hull area, compactness, eccentricity, object extent X, and object extent Y of the MGRIL group increased, and only the convex hull area decreased slightly at 9W. All parameters showed continuous distributions at each development stage.

**Figure 6:**
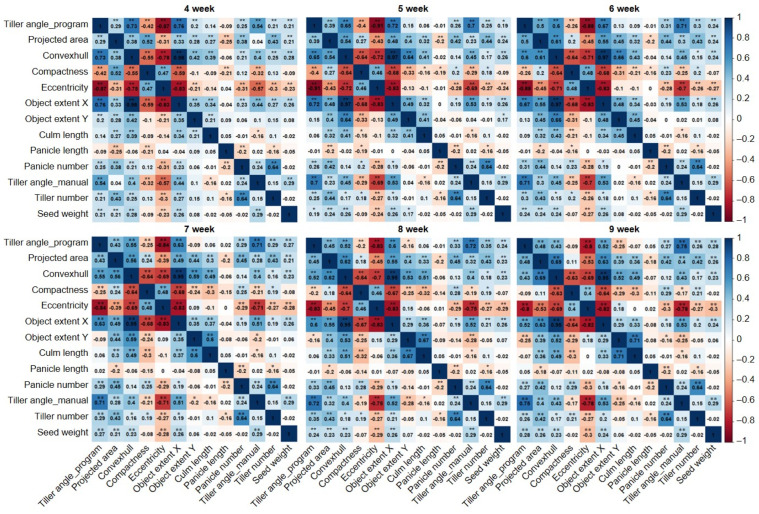
Correlation plots between tiller angle, RGB parameters, and manually measured agronomic traits. The correlation coefficient (*r*-value) was calculated from the mean value of each trait across six development stages. The intensity of the color indicates the strength of the correlation, with +1 indicating a strong positive correlation between the two traits (dark blue) and −1 indicating a strong negative correlation (dark red). W, weeks after sowing (development stage). * significant at the 0.05 level. ** significant at the 0.01 level.

In the correlation analysis between tiller angle, image-based parameters, and manually measured major agronomic traits, the tiller angle measured by the program showed a significant correlation with the manually measured tiller angle (Figure 6). The correlation coefficient (*r*-value) increased from 0.54 at 4W to 0.75 at 9W, and gradually increased with the development stage. Among the image-based parameters, eccentricity (*r*-value: −0.91–−0.8), object extent X (0.52–0.76), and convex hull area (0.43–0.73) had significantly strong correlations with the tiller angle, and eccentricity showed the highest negative correlation in all development stages. Projected area (0.29–0.5), compactness (−0.42–−0.09), panicle number (0.25–0.31), tiller number (0.21–0.35), and seed weight (0.19–0.28) showed weak correlations with tiller angle. Object extent Y showed almost no correlation, and culm length and panicle length showed no correlation.

### 2.4. Unconditional QTLs for Tiller Angle and Various Agronomic Traits of Rice

**Figure 7:**
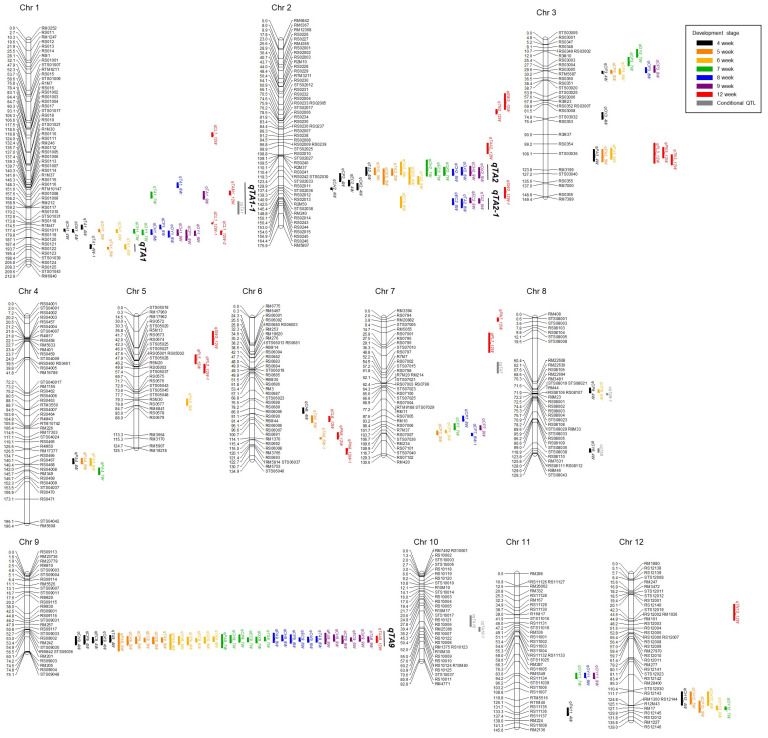
Chromosomal locations of QTL associated with tiller angle and 12 agronomic traits. A total of 136 unconditional QTLs and 6 conditional QTLs were detected in Milyang23/Giho RIL population. The bars representing QTL were created by the confidence interval of the QTL and were represented in different colors for each development stage.

QTL mapping was performed for each development stage, and the significant threshold was set at LOD > 3.0. A total of 136 QTLs for tiller angle and its related agronomic traits were identified from 11 rice chromosomes, except chromosome 10 (Figure 7 and Figure S1, Tables S2–S9). Among them, 20 QTLs associated with tiller angles measured by the program were detected on chromosomes 1, 2, 6, and 9. Specifically, major QTLs *qTA1* (*qTA1_4W-1*, *qTA1_5W*, *qTA1_6W*) and qTA1-1 (*qTA1_7W*, *qTA1_8W*, *qTA1_9W*) that overlapped at various development stages were detected on chromosome 1, *qTA2* (*qTA2_4W*, *qTA2_5W*, *qTA2_6W*) and *qTA2*-1 (*qTA2_8W*, *qTA2_9W*) were detected on chromosome 2, and *qTA9* (*qTA9_4W*, *qTA9_5W*, *qTA9_6W*, *qTA9_7W*, *qTA9_8W*, *qTA9_9W*) was detected on chromosome 9. The LOD values of five QTLs for tiller angle, *qTA1*, *qTA1-1*, *qTA2*, *qTA2-1*, and *qTA9*, were 5.1–5.9, 3.8–4.7, 4.7–8.2, 4.6–4.9, and 13.0–22.74, respectively, explaining 7.24–7.94%, 5.46–8.15%, 7.33–11.45%, 6.98–7.31%, and 23.34–42.13% of the phenotypic variation explained (PVE), respectively. The LOD and PVE values of *qTA1*, *qTA2*, and *qTA9* were highest in 5W. Positive alleles of 12 QTLs in *TA1*, *qTA1-1*, and *qTA9* were derived from Milyang23, and positive alleles of 5 QTLs in *qTA2* and *qTA2-1* were derived from Giho. In *qTA2*, QTLs associated with convex hull area, eccentricity, and object extent X were detected at all development stages, while QTL for compactness was detected only at 4W. *qTA9*, which had the highest LOD value, was located in RM257-STS09048, and in that region, 36 QTLs associated with projected area, convex hull area, compactness, eccentricity, object extent X, and manually measured tiller angle were detected at all development stages. Therefore, *qTA9* can be considered a major region controlling the tiller angle and plant type of rice.

### 2.5. Conditional QTLs for Tiller Angle

A total of six conditional QTLs for tiller angle were detected on chromosomes 1, 7, 8, and 10, with individual LOD values of 3.6–4.1 which explained 6.92% to 9.59% of the PVE (Figure 7, Table S10). These QTLs consisted of one consensus QTL overlapping with the unconditional QTLs for tiller angle measured by the program, and five non-consensus QTLs. Three QTLs were identified for ∆T2, representing changes between 4W and 5W. *cqTA1* overlapped with *qTA1*-1 and showed an additive effect in the same direction. *cqTA7* did not overlap with any unconditional QTL, and *cqTA8* overlapped with qOY8_4W but exhibited an opposite additive effect. One QTL was identified for ∆T3, representing changes between 5W and 6W. *cqTA8*-1 overlapped with qCP8_4W and qCP8_6W, showing the same direction of additive effect, and also exhibited the highest LOD value of 4.1 among the conditional QTLs. Two QTLs, *cqTA10* and *cqTA10*-1, were identified for ∆T5, representing changes between 7W and 8W, and did not overlap with any unconditional QTL. No QTLs were identified for ∆T4 and ∆T6.

In conclusion, comparing the number of QTLs related to tiller angle, QTLs detected by the unconditional QTL mapping method accounted for 79% of the total, with the majority detected at 4W, 5W, and 6W, and QTLs detected by the conditional QTL mapping method were also detected most in 4–6W (Figure S2).

### 2.6. Gene Ontology Analysis of Candidate Genes Associated with Tiller Angle

**Figure 8:**
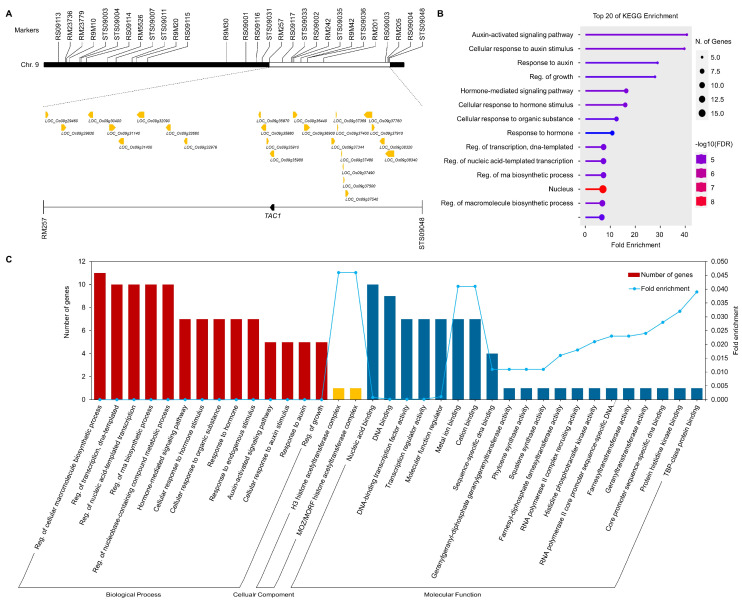
Functional analysis of 26 candidate genes related to tiller angle. (**A**) Physical map of 26 candidate genes in RM257-STS09048 on chromosome 9. (**B**) Gene Ontology (GO) enrichment and KEGG pathway analysis of 26 candidate genes associated with tiller angle. (**C**) The top-enriched GO terms of candidate genes (*p* < 0.05) in the categories of Biological Process, Cellular Component, and Molecular Function. Fold enrichment represents the background frequency of the total genes annotated with a term compared to the number of input genes corresponding to the same term.

The marker interval of the RM257-STS09048 region on chromosome 9 is 27.2 cM, and the open reading frames (ORFs) between these markers were screened to search for candidate genes related to tiller angle. A total of 385 genes were identified in this region, and 26 candidate genes related to tiller angle were selected according to the gene function databases provided by RiceXpro, RGAP, and NCBI (Figure 8A, Table S11). Among them, *LOC_Os09g35980* is well known as *TILLER ANGLE CONTROL 1* (*TAC1*) gene, which regulates tiller angle and horizontal shoot growth. Gene ontology analysis was performed to systematically analyze the functions of the selected candidate genes (Figure 8B,C). From a set of 14 terms related to biological processes, 2 terms for cellular components, and 20 terms for molecular functions, 14 top-ranked GO terms were selected across all possible gene sets. The most enriched GO terms for all possible gene sets and biological processes were auxin signaling, growth regulation, and hormone signaling. In the cellular component, histone acetyltransferase complex was identified, and in the molecular function, nucleic acid binding, DNA binding, and transcription regulator activity were identified.

## 3. Discussion

The architecture of rice is a key factor that supports and maintains high yields by efficiently utilizing limited resources such as water, nutrients, and sunlight in unstable environments. Various challenges facing agriculture, such as climate change and lack of arable land, have further highlighted the importance of optimizing the structural characteristics of rice, such as tiller angle [23]. Therefore, exploring novel genes controlling tiller angle plays an important role in increasing rice yield, ensuring sustainable agriculture and food security. The variation in tiller angle has been explained more by genetic effects than by environmental factors [24]. Photoperiod and temperature, as external environmental factors, have been reported to influence tiller angle. However, these effects are season-specific [5,25]. The application of image-based phenotyping technology to the study of tiller angle can facilitate the explanation of genetic effects through precise phenotypic measurements under controlled environmental conditions and generate reproducible results that enhance the reliability of QTL mapping. In our study, we precisely measured tiller angle using RGB images and analyzed plant traits related to tiller angle in a time series using several image-based parameters, including projected area, convex hull area, compactness, and center of mass X (Figure 1, Figure 2 and Figure 3).

In general, *indica* and *japonica* rice are mainly distinguished by morphological characteristics. In the case of tiller angle, *indica* rice shows a wide plant type, and japonica rice shows a compact plant type [26]. In the case of the parent cultivars of the MGRIL population, “Milyang23”, *indica*-type rice, showed a larger tiller angle than the Giho, *japonica*-type rice. In the MGRIL population, 16% of the population had a larger tiller angle than Milyang23, and 25% had a lower tiller angle than Giho, which showed sufficient variation within the population and was suitable for QTL mapping (Figure 4). We measured tiller angle, image-based parameters, and manually measured agronomic traits, and performed correlation analysis (Figure 5 and Figure 6). According to the results, the correlation between the program-measured (during 4–9W) and manually measured (at 12W) tiller angles showed a significant positive correlation. However, the correlation coefficient at 4W was low at 0.54, which may be because the tiller was not fully formed in 4W. The correlation coefficients of 5–9W were 0.70–0.75. Tillering occurs 3-dimensionally (3D), but since measurements from camera images are made 2D, the measured angle may be distorted depending on the camera angle [27]. Therefore, there may be differences between image-based and actual tiller angle due to differences in plant height and tiller position, and methods such as collecting data from various camera angles are necessary to increase accuracy.

Among image-based parameters, the most effective indicators of tiller angles were eccentricity, object extent X, and convex hull area. Eccentricity is a value that represents the characteristics of a conic section. The closer the value of eccentricity is to 0, the more circular the plant is, and the closer it is to 1, the more linear the plant is. As the tiller angle increases, the wider the plant becomes, which is closer to a circular shape. Therefore, the tiller angle and eccentricity have a negative correlation. On the other hand, the object extent X, which represents the horizontal length of the plant, and the convex hull area, which represents the area of the outer outline of the plant, increase, which is consistent with the correlation analysis results. At 9W, the convex hull area decreased for a while, but this may be due to the structural stabilization of the rice, and it still maintained a positive correlation with the tiller angle [7]. The integration of image-based parameters associated with specific traits can significantly enhance the efficiency and precision of breeding programs [28]. Particularly for traits that are challenging to assess subjectively, such as color or stress responses, these parameters facilitate accurate and consistent measurements, providing an opportunity for more refined and robust trait comparisons [29,30]. Morphological traits, such as tiller angle, can also be efficiently assessed using image-based parameters, enabling the rapid identification of phenotypic variation among individuals and allowing for targeted selection aligned with breeding objectives. Moreover, leveraging image-based parameters for QTL and GWAS (Genome-Wide Association Study) analyses can improve the accuracy and reliability of these approaches. If the selected image parameters exhibit strong associations with trait variation, they can enhance the detection of overlapping genomic regions in QTL mapping, increasing the likelihood of identifying loci that play a significant role in trait determination. QTLs that are consistently detected in a specific genetic region can be considered as reliable loci that have been repeatedly verified [31,32].

In this study, we performed dynamic QTL mapping for tiller angle. We identified five major unconditional QTLs associated with tiller angle on chromosomes 1, 2, and 9 with LOD ≥ 4.7 and named them *qTA1*, *qTA1-1*, *qTA2*, *qTA2-1*, and *qTA9* (Figure 5, Table S2). Among the six identified conditional QTLs, *cqTA1* was a consensus QTL with *qTA1-1*. The detection of QTLs related to tiller angle showed various patterns depending on the development stage. Unconditional and conditional QTLs for tiller angle were detected on multiple chromosomes and locations depending on the development stage, and were most frequently detected at 4–6W (Figure S2). In particular, *qTA1*, *qTA2*, and *qTA9* showed the highest LOD values at 5W, and *cqTA8-1*, which had the highest LOD value among the conditional QTLs, was also detected at 5–6W. Tiller development continues through the “early spread stage” up to 60 days after sowing (DAS), during which the tiller angle increases in the initial spread stage and reaches its widest at 60 DAS. Therefore, QTLs detected at this stage may be key regulators of tiller angle [7]. Therefore, our results indicate that the region identified at 5W may be an important locus affecting the tiller angle.

Although conditional QTL for tiller angle has not been reported, research on unconditional QTL has progressed significantly. Many previous studies have identified QTLs associated with tiller angle on chromosomes 1, 2, and 9. In 238 micro-core germplasm populations, a genome-wide association analysis (GWAS) identified QTLs on chromosomes 1, 2, and 9 with PVE of 5.94–12.38% over 2 years [24]. In the RIL populations of Lemont and Yangdao4, *qTA1*, *qTA2.1*, and *qTA9* were detected more than three times, and among them, only *qTA9* showed a large PVE of more than 10% [33]. Four QTLs (*qTA1.1*, *qTA7.1*, *qTA9.1* and *qTA2.1*) were significantly identified in the chromosome segment substitution lines of wild rice DP30 [34]. Therefore, it is thought that genes located on chromosomes 1, 2, and 9 may interact with each other and cause large variations in tiller angle. The additive effect values of QTLs show that positive alleles of *qTA1*, *qTA1-1*, and *qTA9* derived from the wide plant type, Milyang23. This suggests that the genes in the QTLs may be genes that positively regulate tiller angle. *TAC1*, which was identified in the tiller angle control QTL on chromosome 9 in a previous study, is a gene that regulates the increasing of the tiller angle. That is, when the expression of *TAC1* increases, the tiller of the rice grows closer to horizontal [35,36,37,38]. In contrast, *qTA2* and *qTA2-1* can negatively regulate the tiller angle. In addition, minor QTLs *qTA1_4W* (LOD: 3.4, PVE: 4.96%), *qTA6_5W* (3.4, 4.96%), and *qTA6_5W-1* (4.9, 6.14%) can affect the tiller angle in combination with the major QTLs. QTLs associated with convex hull area, eccentricity, and object extent X, which were parameters correlated with tiller angle, were also intensively identified on chromosomes 1, 2, and 9 (Figure 5, Tables S3–S9). These results support that these traits are suitable as indicators for exploring tiller angle.

A total of 26 candidate genes related to tiller angle were screened and GO analysis was conducted to better understand the functions and pathways of the candidate genes (Figure 8, Table S11). GO enrichment results related to biological processes showed a significant overrepresentation of genes involved in auxin signaling related to tiller angle. GO terms such as “Auxin-activated signaling pathway (GO:0009734)” and “cellular response to auxin stimulus (GO:0071365)” indicate that tiller angle is likely regulated through an auxin-related hormone regulatory mechanism. This supports previous studies that tiller angle is regulated through the asymmetric auxin pathway in rice, maize, and arabidopsis [13,39,40,41]. GO terms related to cellular components were not prominent, but GO terms related to “Histone acetyltransferase (GO:0070775, GO:0070776)”, which regulates gene transcription and plant growth, were found [42]. Among the molecular functions involved in tiller angle, the abundance of genes related to hormone biosynthesis was highlighted. For example, “geranylgeranyl-diphosphate geranylgeranyltransferase activity (GO:0016767)” and “phytoene synthase activity (GO:0046905)” were significantly overrepresented. Phytoene is a precursor for gibberellin and carotenoid biosynthesis, suggesting again that hormone regulation is involved in tiller angle [43]. Among the candidate genes, *TAC1* (*LOC_Os09g35980*), a well-known regulator of tiller angle, was identified. To investigate the effect of the *TAC1* gene in the MGRIL population, we compared the *TAC1* sequence of Milyang23 and Giho (Figure S3). We found that in the third exon region of *TAC1*, the nucleotide sequence of Milyang23 was ‘ACG’, while in Giho it was ‘ACA’. Additionally, in the splicing site of the 3′-untranslated region (3′-UTR), the nucleotide sequence of Milyang23 was ‘AGGA’, while in Giho it was ‘GGGA’. According to Yu et al., a mutation from ‘AGGA’ to ‘GGGA’ in the fourth intron of the 3′-UTR region of the *TAC1* gene causes a splicing failure, reducing *TAC1* levels, which results in a compact plant structure with a tiller angle close to zero [7]. This supports the idea that the narrow tiller angle in Giho is due to this mutation, further confirming that *TAC1* is a key regulator of tiller angle in rice.

## 4. Materials and Methods

### 4.1. Plant Materials

For the identification of QTL associated with tiller angle, 162 recombinant inbred lines (MGRILs) derived from the cross between ‘Milyang23’ (Tongil type) and ‘Giho’ (*Japonica* type) were used as plant materials. MGRILs is a population that was first crossed in 1988 and fixed for more than F_25_. Seeds were disinfected with a fungicide, soaked at 28°C for 4 days, and grown in a greenhouse for 2 weeks. Pots (15 cm in diameter) were filled with 600 g of soil and saturated with water for 3 days. Seedlings were transplanted into the pots and then transported to a conveyor system. Environmental conditions in the greenhouse were maintained under a photoperiod of 14 h light/10 h dark, humidity of 50%, and light intensity of 1000 µmol/m^2^. Each line was planted and evaluated in six replicates.

### 4.2. RGB Imaging for Image-Based Parameter Extraction

From 4W to 9W, RGB images of Milyang23, Giho, and MGRIL population were acquired at 7-day intervals using a 3D scanalyzer imaging system (LemnaTec, GmbH, Aachen, Germany). A camera with a resolution of 6576 × 4384 was fixed at a consistent position within the chamber and set to exposure: 26,000 µs, gain: 150, gamma: 75. RGB images were captured from three angles (0°, 120°, and 240°) and automatically stored on a server. The accumulated images were converted to PNG files and analyzed using LemnaGrid software V7.8.0 (LemnaTec, GmbH, Aachen, Germany). The algorithm for background separation and morphological data acquisition was developed using a Matlab program V7.2 (MathWorks, Natick, MA, USA, https://www.mathworks.com/products/matlab.html, accessed on 1 December 2017) [44]. The acquired images were converted to Lab and HSI channels for easier separation of the region of interest (ROI) and background. The converted images were binarized using thresholds applied to the a* and b* channels of the LAB space, and the H channel of the HSI space, to remove the background [15]. To enhance the quality of crop images after background removal, the median filter method was used, with a 9 × 9 square mask. The morphology technique was applied to correct the damaged areas in the crop images [45]. To analyze the morphological characteristics of the crop, the original and binarized images were processed using the masking technique to separate the region of interest. Six image parameters—projected area, convex hull area, compactness, eccentricity, object extent X, and object extent Y—were extracted. The definition of each image parameter is described in Table S12 [46].

### 4.3. Measurement of Tiller Angle Using RGB Images

The tiller angle was defined as the maximum angle between the outermost tillers of the plant, and measured using a tiller angle measurement program developed by the National Institute of Agricultural Sciences, Rural Development Administration. RGB images obtained from the 3D scanalyzer imaging system from 4–9W were used to measure the tiller angle. The ROI image obtained through background removal and binarization is segmented from the bottom of the plant to a certain height, and then coordinates are assigned to the top and bottom vertices of the plant. The tiller angle is obtained based on the size of the internal angle calculated by applying the atan function to the length of the side connecting each coordinate.

### 4.4. Measurement of Major Agronomic Traits of Rice

The main agronomic traits of rice, such as culm length, panicle length, panicle number, tiller angle, tiller number, and seed weight were measured at 12W. The tiller angle was measured using a laser distance meter (GLM-50C, Bosch, Gerlingen, Germany), and the seed weight was measured by weighing 100 seeds dried after harvest. All traits were measured six times.

### 4.5. Construction of Genetic Map

QTL analysis was performed using the tiller angle measured by the program, six image-based parameters, and six manually measured agricultural traits. The genetic map consisted of 466 DNA markers consisting of Indel, RTM, STS, and SSR markers, and the total genetic distance was 1697 cM. MapDisto 1.7.7 was used to construct the genetic map of the population, and the linkage distance between markers was calculated by the Kosambi method [47]. 

### 4.6. Dynamic QTL Analysis

Unconditional phenotypic values for QTL mapping were based on tiller angle and image-based parameters measured from 4–9W, and agronomic traits measured at 12W. Conditional phenotypic values were the increments in tiller angle measured by the program at adjacent stages: ΔT2 (5W-4W), ΔT3 (6W-5W), ΔT4 (7W-6W), ΔT5 (8W-7W), and ΔT6 (9W-8W). The conditional phenotypic value ΔT1, corresponding to the phenotype at 4W, was considered the unconditional genetic effect for 4W, as it reflected the 4W stage’s phenotypic value [48,49]. QTL mapping was performed using Windows QTL Cartographer V2.5 (Department of Statistics, North Carolina State University, Raleigh, NC, USA). The CIM (Composite Interval Mapping) method was used for QTL analysis, and the LOD threshold for determining the presence of significant QTL was determined by a permutation test with 1000 repetitions (*p* < 0.05) [50]. QTL naming followed the McCouch method [51]. The genetic linkage map and graphical presentation of QTLs were created using MapChart 2.32 software [52].

### 4.7. Gene Ontology Analysis of Candidate Genes

The chromosomal locations of markers for searching candidate genes were searched in Rapdb (https://rapdb.dna.afrc.go.jp/ accessed on 17 July 2024). Screening of open reading frames (ORFs) between markers and gene function analysis were performed using RiceXpro (https://ricexpro.dna.afrc.go.jp/ accessed on 17 July 2024), Rice Genome Annotation Project (RGAP, https://rice.uga.edu/ accessed on 23 July 2024), NCBI (https://www.ncbi.nlm.nih.gov/ accessed on 23 July 2024), and STRING 12.0 (https://string-db.org/ accessed on 23 July 2024). GO enrichment analysis of the candidate genes was performed using the ShinyGO 0.80 (http://bioinformatics.sdstate.edu/go/ accessed on 2 August 2024) online tool.

### 4.8. Multiple Sequence Alignment

The genomic sequence and coding sequence (CDS) of the candidate gene (Gene ID: 4347655) were obtain from NCBI (https://www.ncbi.nlm.nih.gov/ accessed on 9 January 2020), and the genomic DNA of Milyang23 and Giho for sequencing was extracted by the cetyltrimethylammonium bromide (CTAB) method [53]. Multiple sequence alignment was performed using the CLC Genomics Workbench Program (ver. 8.0, Qiagen, Aarhus, Denmark).

### 4.9. Statistical Analysis

The significance of the difference in tiller angle was analyzed using the *t*-test (*p* < 0.05) in SPSS 22.0 (IBM SPSS Statistics, Armonk, NY, USA). The correlations among tiller angle, image-based parameters, and agronomic traits were analyzed using the “corrplot” package in R version 4.3.1 (R Foundation for Statistical Computing, Vienna, Austria), and the significance of the correlation coefficient was analyzed using Pearson’s correlation coefficient (*p* < 0.05).

## 5. Conclusions

In this study, we measured the tiller angle of rice using an image-based phenotyping technique to overcome the limitations of existing manual and labor-intensive phenotypic analysis methods, and explored QTLs related to tiller angle. As a result, the accuracy of the automatically measured tiller angle using RGB images was 75%, and eccentricity was confirmed to be the parameter with the highest correlation with tiller angle. Five major QTLs associated with tiller angle were identified on chromosomes 1, 2, and 9, and among them, 26 candidate genes related to auxin signaling and plant growth, including the *TAC1* gene on chromosome 9, were identified. The results of this study suggest a strategy to improve the accuracy of QTL analysis and breeding efficiency using image-based phenotypic analysis. In particular, auxin-related genes including the *TAC1* gene can be utilized as major targets for tiller angle control, and can contribute to improving the plant architecture of rice and increasing agricultural productivity.

## Data Availability

Data are contained within the article and the Supplementary Materials.

## Supplementary Materials

The following supporting information can be downloaded at: https://www.mdpi.com/article/10.3390/plants13233288/s1, Figure S1: Quantitative trait loci (QTL) analysis associated with tiller angle in rice using RIL population; Figure S2: QTL distribution for tiller angle across different development stages. (A) Pie plot showing the percentage of QTL category. (B) The number of unconditional and conditional QTLs for tiller angle at different development stages. QTLs for Tiller angle_program and Tiller angle_manual belong to unconditional QTL; Figure S3: The identification of *TAC1*. (A) Schematic representation of *TAC1* gene structure and DNA sequence comparison between Milyang23 and Giho. Mutations in exon and splicing site of the intron in 3’-untranslated region (UTR) are shown in red. White box, exon; thin line, intron; black box, 3’-UTR; ATG, start code; TAA, stop code. (B) Multiple sequence alignment of *TAC1* gene sequence. The site where the mutation occurred is marked with a red box; Table S1: Tiller angle (°) according to developmental stage of rice; Table S2: List of QTLs for tiller angle measured by image-based programs; Table S3: List of QTLs for projected area in RIL population; Table S4: List of QTLs for convex hull area in RIL population; Table S5: List of QTLs for compactness in RIL population; Table S6: List of QTLs for eccentricity in RIL population; Table S7: List of QTLs for object extent X in RIL population; Table S8: List of TLs for object extent Y in RIL population; Table S9: List of QTLs for agronomic traits in RIL population; Table S10: List of conditional QTLs for tiller angle in RIL population; Table S11: Candidate genes from QTL associated with tiller angle in chromosome 9; Table S12: Image-based parameters used for QTL analysis related to tiller angle.
