# Supplementary material for: Application of Image-Based Phenotyping for QTL Identification of Tiller Angle in Rice (Oryza sativa L.)
Source: Plants (Basel). 2024 Nov 22;13(23):3288. doi: 10.3390/plants13233288 (PMC11644284; doi:10.3390/plants13233288)
Supplement: Supplementary file 1 [file plants-13-03288-s001.zip › plants-3265524-supplementary.pdf]

Supplementary Materials

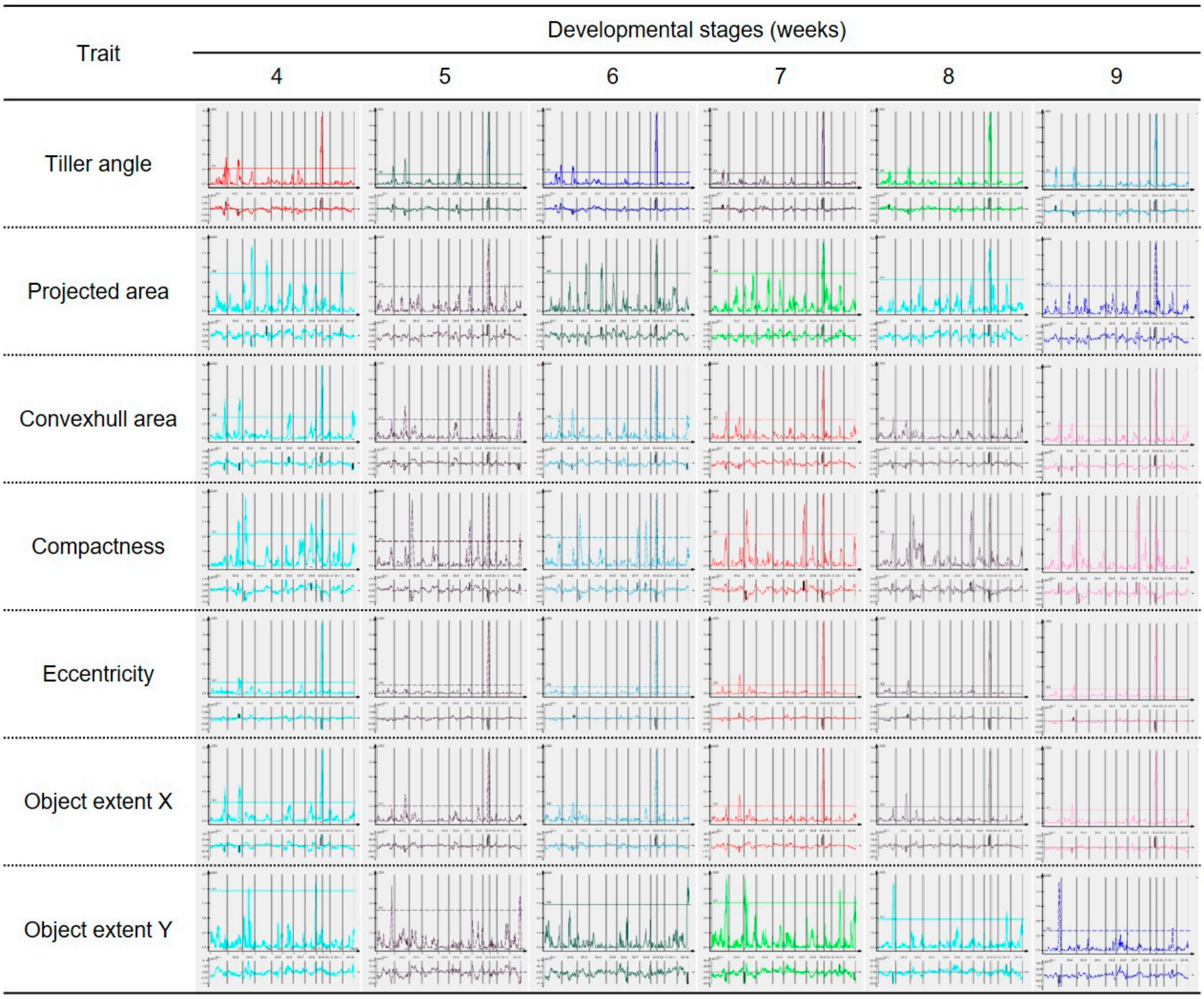

Figure S1. Quantitative trait loci (QTL) analysis associated with tiller angle in rice using RIL population.

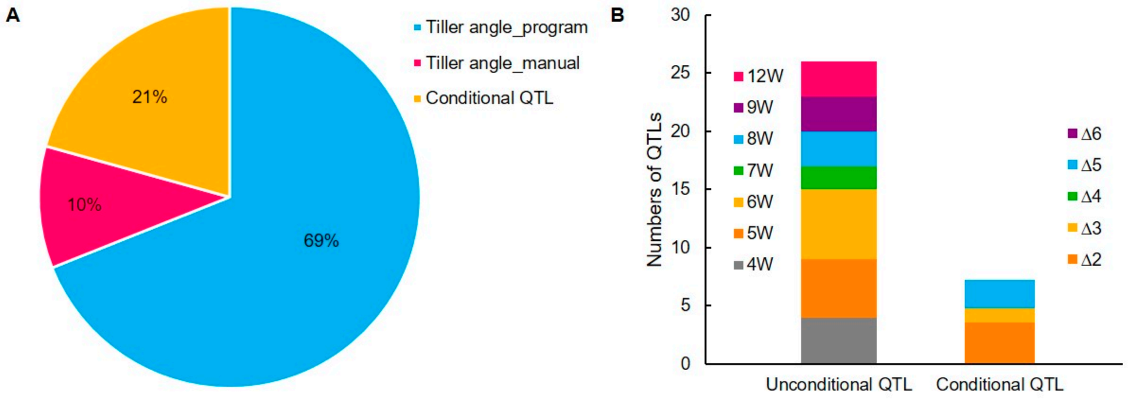

Figure S2. QTL distribution for tiller angle across different development stages. (**A**) Pie plot showing the percentage of QTL category. (**B**) The number of unconditional and conditional QTLs for tiller angle at different development stages. QTLs for Tiller angle_program and Tiller angle_manual belong to unconditional QTL.

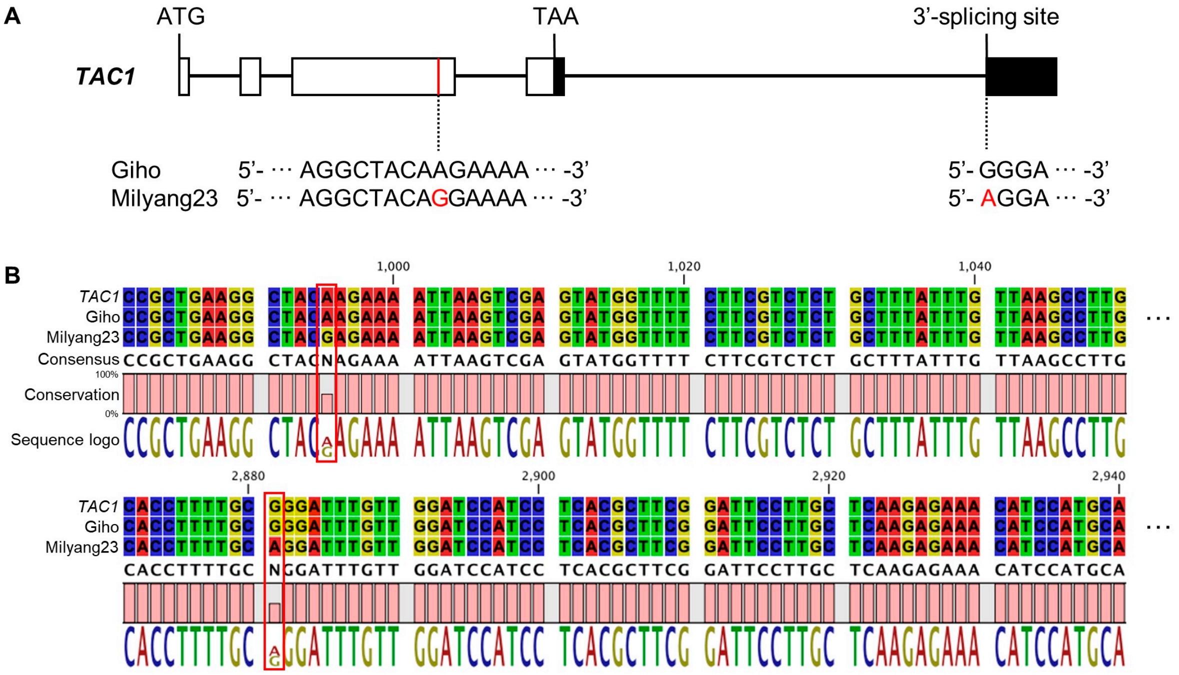

Figure S3. The identification of *TAC1*. (A) Schematic representation of *TAC1* gene structure and DNA sequence comparison between Milyang23 and Giho. Mutations in exon and splicing site of the intron in 3'-untranslated region (UTR) are shown in red. White box, exon; thin line, intron; black box, 3'-UTR; ATG, start code; TAA, stop code. (B) Multiple sequence alignment of *TAC1* gene sequence. The site where the mutation occurred is marked with a red box.

Table S1. Tiller angle (°) according to developmental stage of rice.

Development stage (weeks)	Milyang23	Giho		MGRILs
	Mean±SD[z]		p-value	Mean±SD
4	62.28±6.21[z]	46.81±4.08	<0.001	50.49±2.7
5	56.62±5.73	38.12±5.16	<0.001	41.29±2.48
6	50.86±6.04	31.15±4.82	<0.001	36.71±2.34
7	46.15±4.72	30.19±3.06	<0.001	35.37±2.46
8	44.39±5.71	22.10±5.97	<0.001	31.75±2.79
9	43.19±10.61	24.37±5.55	0.006	28.75±3.11

[z] The data are presented as the mean ± standard deviation (SD).

Table S2. List of QTLs for tiller angle measured by image-based programs.

Development stage (weeks)	QTL	Chromosome	Position (cM)[z]	LOD[y]	Additive effect[x]	R^{2v}	LOD1_L (cM)	LOD1_R (cM)
4	*qTA1_4W*	1	1.7741	3.4	-3.32[w]	4.96	1.752	1.795
	qTA1_4W-1	1	1.9471	5.1	4.18	7.94	1.937	1.980
	qTA2_4W	2	1.2441	4.8	-3.43	7.50	1.201	1.283
	qTA9_4W	9	0.6121	13.0	5.94	23.34	0.588	0.655
5	*qTA1_5W*	1	1.9841	5.8	2.79	7.41	1.962	1.984
	qTA2_5W	2	1.2541	8.2	-3.43	11.45	1.217	1.284
	qTA6_5W	6	0.9511	3.7	2.54	4.57	0.93	0.998
	qTA6_5W-1	6	1.1411	4.9	-2.83	6.14	1.137	1.155
	qTA9_5W	9	0.6321	22.7	6.58	42.13	0.61	0.657
6	*qTA1_6W*	1	1.9741	5.4	2.97	7.24	1.937	1.984
	qTA2_6W	2	1.3291	4.7	-2.61	7.33	1.289	1.369
	qTA9_6W	9	0.6221	19.3	5.81	34.95	0.599	0.649
7	*qTA1_7W*	1	1.4731	4.0	2.33	5.81	1.44	1.504
	qTA9_7W	9	0.6221	19.9	5.80	37.94	0.602	0.645
8	*qTA1_8W*	1	1.3711	3.8	2.19	5.46	1.354	1.404
	qTA2_8W	2	1.5141	4.9	-2.42	6.98	1.501	1.590
	qTA9_8W	9	0.6221	19.6	5.58	36.46	0.595	0.656
9	*qTA1_9W*	1	1.4731	4.7	2.93	8.15	1.435	1.513
	qTA2_9W	2	1.5041	4.6	-2.66	7.31	1.500	1.545
	qTA9_9W	9	0.6221	16.8	5.81	34.54	0.600	0.656

[z] Marker position in cM on chromosome.

[y] Significance threshold of LOD = 3.0.

[x] Additive effect = (Mean of the lines carrying the Milyang23 allele - mean of the lines carrying the Giho allele)/2.

[w] The negative values of the additive effect indicate that alleles from Giho are in the direction of increases in the traits.

[v] The proportion of evaluated phenotype variations attributable to a particular QTL was estimated using the coefficient of determination.

Table S3. List of QTLs for projected area in RIL population.

Development stage (weeks)	QTL	Chromosome	Position (cM)	LOD	Additive effect	R^2	LOD1_L (cM)	LOD1_R (cM)
4	*qPA3_4W*	3	1.0811	5.4	-14.04	10.40	1.043	1.157
	qPA4_4W	4	1.3771	4.2	13.34	9.50	1.347	1.401
	qPA11_4W	11	1.3111	3.5	11.29	6.70	1.27	1.346
5	*qPA3_5W*	3	1.0811	3.8	-20.60	7.70	1.043	1.164
	qPA4_5W	4	1.3771	3.6	21.54	8.60	1.346	1.401
	qPA9_5W	9	0.6621	3.6	21.96	9.00	0.565	0.742
6	*qPA3_6W*	3	1.0811	4.0	-27.19	8.00	1.028	1.139
	qPA4_6W	4	1.3771	3.6	27.25	8.40	1.346	1.42
	qPA5_6W	5	0.7991	3.1	24.16	6.20	0.783	0.847
	qPA9_6W	9	0.6621	5.5	35.76	14.20	0.578	0.724
7	*qPA4_7W*	4	1.4671	3.2	28.29	6.50	1.436	1.503
	qPA9_7W	9	0.6421	5.7	42.16	14.40	0.578	0.711
8	*qPA9_8W*	9	0.5851	6.1	47.74	13.20	0.565	0.659
9	*qPA9_9W*	9	0.5851	7.7	59.43	16.30	0.565	0.672

Table S4. List of QTLs for convex hull area in RIL population.

Development stage (weeks)	QTL	Chromosome	Position (cM)	LOD	Additive effect	R^2	LOD1_L (cM)	LOD1_R (cM)
4	*qCH1_4W*	1	1.7981	6.0	-1.80	8.80	1.791	1.816
	qCH2_4W	2	1.4031	6.1	-1.82	9.40	1.396	1.418
	qCH6_4W	6	0.8701	3.9	1.52	5.90	0.854	0.91
	qCH9_4W	9	0.6321	11.0	2.72	20.60	0.596	0.682
	qCH12_4W	12	1.1781	4.2	-1.66	7.90	1.117	1.248
5	*qCH2_5W*	2	1.2441	5.3	-2.13	9.40	1.208	1.278
	qCH9_5W	9	0.6321	11.3	3.38	23.20	0.596	0.683
	qCH12_5W	12	1.1781	4.4	-2.13	9.30	1.118	1.24
6	*qCH1_6W*	1	1.7981	4.1	-1.94	6.40	1.794	1.829
	qCH2_6W	2	1.2131	4.4	-2.03	7.50	1.151	1.278
	qCH9_6W	9	0.6321	11.0	3.57	22.90	0.597	0.678
	qCH12_6W	12	1.1881	3.5	-2.07	7.80	1.118	1.252
7	*qCH1_7W*	1	1.7981	4.4	-2.27	7.20	1.794	1.833
	qCH2_7W	2	1.2331	3.5	-1.98	5.70	1.134	1.289
	qCH9_7W	9	0.6221	11.2	4.03	23.30	0.587	0.664
8	*qCH1_8W*	1	1.8081	3.9	-2.49	7.10	1.795	1.847
	qCH2_8W	2	1.2341	3.4	-2.13	5.50	1.222	1.283
	qCH9_8W	9	0.6121	12.6	4.62	25.70	0.582	0.645
9	*qCH1_9W*	1	1.8081	3.8	-2.47	6.80	1.795	1.854
	qCH2_9W	2	1.2341	3.5	-2.17	5.60	1.194	1.287
	qCH9_9W	9	0.6121	13.2	4.76	26.60	0.581	0.646

Table S5. List of QTLs for compactness in RIL population.

Development stage (weeks)	QTL	Chromosome	Position (cM)	LOD	Additive effect	R^2	LOD1_L (cM)	LOD1_R (cM)
4	*qCP2_4W*	2	1.2891	4.4	0.01	7.16	1.262	1.344
	qCP3_4W	3	0.3021	6.6	-0.01	11.11	0.301	0.327
	qCP8_4W	8	0.7031	4.0	0.01	6.49	0.666	0.716
	qCP9_4W	9	0.6221	6.4	-0.01	12.61	0.588	0.675
5	*qCP3_5W*	3	0.3021	8.1	-0.01	13.62	0.292	0.325
	qCP7_5W	7	1.0871	5.7	0.01	9.84	1.073	1.115
	qCP9_5W	9	0.6321	8.6	-0.01	18.94	0.601	0.668
	qCP12_5W	12	1.2481	4.0	0.01	6.33	1.205	1.317
6	*qCP3_6W*	3	0.2961	5.6	-0.01	10.17	0.278	0.333
	qCP7_6W	7	1.0871	4.3	0.01	8.28	1.068	1.096
	qCP8_6W	8	0.6961	4.6	0.01	8.55	0.681	0.713
	qCP9_6W	9	0.6321	7.3	-0.01	17.24	0.603	0.68
7	*qCP3_7W*	3	0.2301	4.3	-0.01	9.84	0.17	0.254
	qCP7_7W	7	1.0371	5.6	0.01	10.27	1.004	1.067
	qCP9_7W	9	0.6121	6.8	-0.01	14.27	0.579	0.657
8	*qCP1_8W*	1	1.7741	3.2	0.01	5.72	1.752	1.794
	qCP3_8W	3	0.2961	5.0	-0.01	9.31	0.266	0.32
	qCP7_8W	7	0.9521	3.1	0.01	7.16	0.931	0.984
	qCP7_8W-1	7	1.0991	4.8	0.01	8.89	1.096	1.124
	qCP9_8W	9	0.6221	6.6	-0.01	15.07	0.58	0.665
9	*qCP1_9W*	1	1.8481	4.5	0.01	9.47	1.8	1.901
	qCP3_9W	3	0.2961	4.2	-0.01	7.97	0.249	0.315
	qCP7_9W	7	1.0371	5.7	0.01	10.98	1.022	1.067
	qCP9_9W	9	0.6121	3.7	-0.01	7.78	0.59	0.67

Table S6. List of QTLs for eccentricity in RIL population.

Development stage (weeks)	QTL	Chromosome	Position (cM)	LOD	Additive effect	R^2	LOD1_L (cM)	LOD1_R (cM)
4	*qEC2_4W*	2	1.3291	4.5	0.05	7.67	1.325	1.384
	qEC9_4W	9	0.6321	19.3	-0.12	44.35	0.605	0.662
5	*qEC9_5W*	9	0.6321	26.6	-0.13	55.59	0.611	0.653
6	*qEC2_6W*	2	1.5141	3.5	0.04	4.79	1.479	1.588
	qEC9_6W	9	0.6321	32.1	-0.14	63.91	0.615	0.653
7	*qEC2_7W*	2	1.2441	7.0	0.05	9.45	1.21	1.282
	qEC9_7W	9	0.6321	26.1	-0.12	49.85	0.609	0.652
8	*qEC2_8W*	2	1.5141	5.4	0.04	6.76	1.499	1.561
	qEC9_8W	9	0.6221	29.7	-0.13	54.93	0.609	0.646
9	*qEC2_9W*	2	1.5041	5.3	0.04	6.47	1.493	1.543
	qEC9_9W	9	0.6221	30.4	-0.12	56.96	0.609	0.648

Table S7. List of QTLs for object extent X in RIL population.

Development stage (weeks)	QTL	Chromosome	Position (cM)	LOD	Additive effect	R^2	LOD1_L (cM)	LOD1_R (cM)
4	*qOX1_4W*	1	1.7981	5.5	-107.39	7.91	1.794	1.824
	qOX2_4W	2	1.4031	6.0	-113.13	9.16	1.354	1.42
	qOX9_4W	9	0.6321	11.6	180.28	22.82	0.596	0.68
5	*qOX2_5W*	2	1.2641	5.5	-104.13	8.96	1.219	1.369
	qOX9_5W	9	0.6421	14.1	194.86	30.61	0.604	0.673
6	*qOX2_6W*	2	1.2641	3.8	-88.20	6.24	1.205	1.369
	qOX9_6W	9	0.6321	14.6	197.38	31.04	0.602	0.666
7	*qOX2_7W*	2	1.2441	5.4	-105.27	8.95	1.202	1.285
	qOX9_7W	9	0.6221	15.0	195.92	30.81	0.603	0.663
8	*qOX2_8W*	2	1.2341	6.1	-108.50	9.32	1.202	1.281
	qOX9_8W	9	0.6221	15.3	198.52	31.04	0.601	0.657
9	*qOX2_9W*	2	1.2541	4.5	-98.04	7.76	1.205	1.31
	qOX9_9W	9	0.6121	16.2	202.27	32.86	0.585	0.647

Table S8. List of TLs for object extent Y in RIL population.

Development stage (weeks)	QTL	Chromosome	Position (cM)	LOD	Additive effect	R^2	LOD1_L (cM)	LOD1_R (cM)
4	*qOY3_4W*	3	0.7481	3.3	13.65	7.82	0.692	0.76
	qOY8_4W	8	1.2581	3.3	12.00	7.78	1.242	1.278
5	*qOY1_5W*	1	1.7981	5.0	-26.68	10.28	1.794	1.82
6	*qOY12_6W*	12	1.2621	4.8	-31.97	11.31	1.252	1.291
7	*qOY1_7W*	1	1.8081	4.7	-49.59	10.11	1.798	1.848
	qOY3_7W	3	0.1071	4.7	-59.51	9.44	0.095	0.173
	qOY11_7W	11	0.9621	3.9	43.40	7.81	0.941	0.996
	qOY12_7W	12	1.2711	3.5	-40.79	6.96	1.261	1.29
8	*qOY1_8W*	1	1.8381	7.1	-82.58	16.34	1.807	1.871
	qOY11_8W	11	0.9621	3.9	55.67	7.59	0.94	0.987
9	*qOY1_9W*	1	1.8281	10.4	-105.60	23.22	1.81	1.855
	qOY11_9W	11	0.9621	3.4	53.26	6.19	0.94	0.997

Table S9. List of QTLs for agronomic traits in RIL population.

Trait	QTL	Chromosome	Position (cM)	LOD	Additive effect	R^2	LOD1_L (cM)	LOD1_R (cM)
Culm length	*qCL1_12W*	1	0.8871	3.8	-3.18	7.53	0.881	0.923
	qCL1_12W-1	1	1.7381	12.2	-6.06	26.64	1.732	1.745
	qCL1_12W-2	1	1.8281	21.6	-7.97	47.86	1.807	1.847
	qCL6_12W	6	0.9611	3.2	2.84	5.61	0.935	0.988
Panicle length	*qPL3_12W*	3	1.0811	2.8	0.76	5.68	0.983	1.177
	qPL5_12W	5	0.4171	3.0	0.78	6.07	0.367	0.453
	qPL5_12W-1	5	0.4981	3.0	0.79	5.95	0.453	0.54
	qPL7_12W	7	0.2601	2.9	0.77	5.83	0.147	0.335
Panicle number	*qPN3_12W*	3	1.0621	8.5	-0.96	18.56	1.023	1.125
	qPN6_12W	6	1.1411	4.2	-0.64	7.89	1.089	1.154
	qPN6_12W-1	6	1.2471	3.4	-0.62	7.50	1.239	1.298
	qPN7_12W	7	0.0301	3.9	0.62	7.54	0.003	0.039
Tiller angle_manual	*qTAM1_12W*	1	1.4631	3.6	1.31	5.63	1.43	1.473
	qTAM2_12W	2	0.9991	3.4	-1.28	5.36	0.975	1.078
	qTAM9_12W	9	0.6221	13.5	2.73	27.46	0.593	0.658
Tiller number	*qTN2_12W*	2	0.6611	3.1	0.57	6.32	0.655	0.701
	qTN3_12W	3	1.1211	3.7	-0.71	9.89	1.09	1.192
	qTN12_12W	12	0.4211	4.4	-0.69	9.05	0.289	0.444
Seed weight	*qSW2_12W*	2	0.6011	3.2	-0.07	6.46	0.506	0.659
	qSW2_12W-1	2	1.4751	3.6	-0.08	7.57	1.392	1.498
	qSW5_12W	5	0.1951	4.8	-0.10	12.21	0.145	0.276

Table S10. List of conditional QTLs for tiller angle in RIL population

Trait	QTL	Chromosome	Position (cM)	LOD	Additive effect	R^2	LOD1_L (cM)	LOD1_R (cM)
Consensus QTL								
ΔT2	*cqTA1*	1	1.5831	3.6	1.55	6.92	1.531	1.653
	cqTA7	7	0.4881	3.9	1.61	7.39	0.423	0.529
	cqTA8	8	1.2581	3.7	-1.65	7.12	1.241	1.278
Non-consensus QTL								
ΔT3	*cqTA8-1*	8	0.7251	4.1	1.10	9.59	0.719	0.735
ΔT5	*cqTA10*	10	0.4631	3.8	1.25	9.42	0.397	0.503
	cqTA10-1	10	0.5201	3.7	1.18	8.53	0.505	0.549

Table S11. Candidate genes from QTL associated with tiller angle in chromosome 9.

Gene	Description
LOC_Os09g29460	Homeodomain leucine zipper protein.
LOC_Os09g29830	Similar to TA1 protein (Fragment).
LOC_Os09g30400	SUSIBA2-like (WRKY transcription factor 80).
LOC_Os09g31140	Similar to T6J4.5 protein (WIP6 protein).
LOC_Os09g31400	Similar to EIL3.
LOC_Os09g32090	Similar to Methyl-CpG-binding domain protein 1 (Methyl-CpG binding protein MBD1).
LOC_Os09g32660	Leucine-rich repeat 2 containing protein.
LOC_Os09g32976	60S ribosomal protein L7a.
LOC_Os09g35870	Similar to Auxin-induced protein IAA4 (OsIAA26).
LOC_Os09g35880	Similar to Hd1-like protein.
LOC_Os09g35910	Similar to Homeodomain leucine zipper protein (Fragment).
LOC_Os09g35980	Conserved hypothetical protein.
LOC_Os09g36440	Transcription initiation factor IIB (General transcription factor TFIIB).
LOC_Os09g36900	Similar to WD-40 repeat protein MSI1.
LOC_Os09g37344	Similar to Auxin induced protein.
LOC_Os09g37369	Similar to Auxin induced protein (OsSAUR41).
LOC_Os09g37400	Auxin responsive SAUR protein family protein.
LOC_Os09g37480	Similar to Auxin induced protein (OsSAUR53).
LOC_Os09g37490	Similar to Auxin induced protein (OsSAUR54).
LOC_Os09g37500	Similar to Auxin induced protein (OsSAUR55).
LOC_Os09g37540	Similar to Lysine decarboxylase-like protein (PA4923).
LOC_Os09g37760	Bromodomain containing protein.
LOC_Os09g37910	Similar to HMGd1 protein (Nucleasome/chromatin assembly factor D protein NFD101).
LOC_Os09g38320	Similar to Phytoene synthase 1, (Fruit ripening specific protein pTOM5).
LOC_Os09g38340	Zinc finger, C2H2-type domain containing protein.
LOC_Os09g39400	Similar to Histidine-containing phosphotransfer protein.

Table S12. Image-based parameters used for QTL analysis related to tiller angle.

Parameter	Description
Projected area	The number of pixels above the object indicating the area of the plant
Convex hull area	The smallest area enclosed by the outer contour of an object
Compactness	The area of an object divided by the convex hull area indicating the density of plants including tillers and leaves
Eccentricity	The degree of roundness of an object. It describes the shape of a conic section, with values ranging from 0 (circle) to 1 (line)
Object extent X	The x-axis length of the rectangle covering the object indicating the plant width
Object extent Y	The y-axis length of the rectangle covering the object indicating the plant height
